# Respiratory Syncytial Virus whole-genome sequencing identifies convergent evolution of sequence duplication in the C-terminus of the G gene

**DOI:** 10.1038/srep26311

**Published:** 2016-05-23

**Authors:** Seth A. Schobel, Karla M. Stucker, Martin L. Moore, Larry J. Anderson, Emma K. Larkin, Jyoti Shankar, Jayati Bera, Vinita Puri, Meghan H. Shilts, Christian Rosas-Salazar, Rebecca A. Halpin, Nadia Fedorova, Susmita Shrivastava, Timothy B. Stockwell, R. Stokes Peebles, Tina V. Hartert, Suman R. Das

**Affiliations:** 1 Infectious Diseases Group, J. Craig Venter Institute, Rockville, MD, USA; 2 Bioinformatics Group, J. Craig Venter Institute, Rockville, MD, USA; 3 Center for Bioinformatics and Computational Biology, University of Maryland, College Park, MD, USA; 4 Division of Infectious Diseases, Department of Pediatrics, Emory University School of Medicine and Children’s Healthcare of Atlanta, Atlanta, GA, USA; 5 Department of Medicine, Vanderbilt University School of Medicine, Nashville, TN, USA; 6 Division of Allergy, Pulmonary, and Critical Care Medicine, Department of Medicine, Vanderbilt University School of Medicine, Nashville, TN, USA; 7 Division of Allergy, Immunology, and Pulmonary Medicine, Department of Pediatrics, Vanderbilt University School of Medicine, Nashville, TN, USA.

## Abstract

Respiratory Syncytial Virus (RSV) is responsible for considerable morbidity and mortality worldwide and is the most important respiratory viral pathogen in infants. Extensive sequence variability within and between RSV group A and B viruses and the ability of multiple clades and sub-clades of RSV to co-circulate are likely mechanisms contributing to the evasion of herd immunity. Surveillance and large-scale whole-genome sequencing of RSV is currently limited but would help identify its evolutionary dynamics and sites of selective immune evasion. In this study, we performed complete-genome next-generation sequencing of 92 RSV isolates from infants in central Tennessee during the 2012–2014 RSV seasons. We identified multiple co-circulating clades of RSV from both the A and B groups. Each clade is defined by signature N- and O-linked glycosylation patterns. Analyses of specific RSV genes revealed high rates of positive selection in the attachment (G) gene. We identified RSV-A viruses in circulation with and without a recently reported 72-nucleotide G gene sequence duplication. Furthermore, we show evidence of convergent evolution of G gene sequence duplication and fixation over time, which suggests a potential fitness advantage of RSV with the G sequence duplication.

Human Respiratory Syncytial Virus (RSV) was first isolated in 1955^1^,^2^,^3^ and has been associated with mild to severe acute lower respiratory tract infections (ALRIs), especially in infants, premature babies, the elderly, and immunocompromised individuals^4^,^5^,^6^,^7^. In 2005, RSV caused an estimated 33.8 million new episodes of ALRIs in children under five worldwide, with 3.4 million cases requiring hospitalization due to severe illness^1^,^7^,^8^. Global estimates of disease burden show RSV to account for 30 million ALRIs and 50,000 annual deaths of children < five years of age^1^,^7^,^8^. Nearly all children have had at least one RSV infection by two years of age^7^. It is well established that RSV infections during infancy (<six months of age) are associated with an increased incidence of subsequent childhood wheezing and asthma^9^. Despite its global public health impact, no licensed vaccines nor effective treatments for acute infection are currently available for RSV^9^. The only approved prophylaxis is passive immunization with palivizumab, a humanized mouse monoclonal antibody against the RSV fusion (F) protein^10^,^11^. The efficacy trials of palivizumab resulted in a 39–78% decrease in hospitalization rates for RSV in premature infants and children with chronic lung disease^10^; however, a recent review shows inconsistent cost-effectiveness of palivizumab^12^.

RSV is an enveloped virus with a negative-sense, single-stranded, non-segmented RNA genome belonging to the *Paramyxoviridae* family. The 11 RSV proteins include the polymerase (L), nucleocapsid (N), phosphoprotein (P), transcriptional regulators (M2-1, M2-2), matrix (M), small hydrophobic protein (SH), non-structural proteins (NS1, NS2) and two major surface glycoproteins (F and G) that are responsible for virus entry and are the major target of human immune responses. The F protein is responsible for the fusion of the viral envelop with the host cell membrane for the viral entry into the cell. The attachment G protein has a short cytoplasmic domain followed by a transmembrane domain and two hypervariable mucin-like domains joined by a conserved sequence, which is responsible for cellular attachment. The G protein also has an immune decoy function in its soluble, extracellularly secreted form.

RSV has an epidemic seasonality similar to the influenza viruses, with increased cases during the winter in temperate climates and during the monsoon season in tropical and sub-tropical climates^1^,^9^,^13^,^14^. RSV can be classified into two antigenic groups (A and B), each containing several distinct subgroups based on antigenic and genomic sequence differences, especially in the G glycoprotein^15^,^16^,^17^. Studies suggest group A viruses cause more severe disease and transmit more readily than group B viruses in infants^9^. These two groups tend to alternate in prevalence between RSV seasons and also show evidence of multiple co-circulating intra-group viral genotypes, or clades, during any given season^9^,^13^,^16^,^18^,^19^, resulting in a diverse set of circulating viruses that can adapt to herd immunity. It is unclear if this represents a gradual evolution of viral genomes or stochastic differences in infection rates by co-circulating strains.

Previous RSV sequencing studies have largely focused on sequencing only complete or partial G gene sequences because the C-terminal, second hypervariable portion of G is sufficient and required for distinguishing the two RSV groups and the various genotypes within each group^13^,^14^. In 1999, a G gene variant was identified in RSV-B that contained a 60-nucleotide (20 amino acid) duplication in the C-terminal third of G, within the second hypervariable mucin-like domain^20^,^21^. This genotype has now spread globally^22^. In 2010, a similar G gene variant was identified in RSV-A from several locations around the globe that contained a 72-nucleotide (24 amino acid) duplication in the second mucin-like domain^15^,^22^,^23^,^24^.

To better understand RSV evolutionary dynamics, we sequenced RSV whole genomes from acutely infected infants from middle Tennessee who were enrolled as part of the *Infant Susceptibility to Pulmonary Infections and Asthma Following RSV Exposure* (INSPIRE) longitudinal observational birth cohort^25^. The objective of our sequencing efforts was to assess the epidemiological and evolutionary dynamics of RSV from Tennessee within a global context, which is important for identifying RSV strains that feed into the global circulation.

## Results

### Large-scale RSV whole-genome sequencing from Nashville, Tennessee

The INSPIRE cohort includes term infants born in June through December such that they are on average less than 6 months of age during the RSV season. The INSPIRE study design included surveillance and collection of nasal wash samples for infants meeting pre-specified respiratory illness criteria occurring from November 1^st^ to March 31^st ^25. A total of 861 nasal wash samples from infants collected in the 2012–2013 season with acute respiratory tract infections were screened for RSV using qRT-PCR. Out of 210 RSV-positive samples, 106 samples from 99 patients were selected for whole-genome sequencing based on disease severity and quality of virus detection by qRT-PCR ct value (<29). Characteristics of these study subjects are in Table 1. Five infants had RSV isolated during two illnesses and one patient had RSV isolated during three illnesses during the 2012–2013 season.

Of the 106 RSV-positive study samples from the 2012–2013 season, 71 RSV whole-genome sequences were obtained, annotated, and submitted to GenBank using an overlapping amplicon-based sequencing approach. Partial genome sequences, obtained from three additional samples that contained gaps and lower coverage areas, were removed from the dataset. In addition, we attempted to sequence 24 RSV-positive samples from the 2013–2014 season, out of which 21 whole-genome sequences were obtained.

### Phylogenetic analyses demonstrate multiple co-circulating RSV lineages in a given season

A maximum likelihood phylogeny that combined the RSV-A and RSV-B lineages was generated using whole-genome sequences from 474 publicly available sequences and the 71 study genomes from the 2012–2013 season (Fig. S1). A subset of these genomes was used to infer a maximum likelihood phylogeny using only the G gene coding sequence, and a similar topology was obtained (Fig. S2). The 71 study genomes segregated into three separate clades: BA RSV-B (seven genomes), GA5 RSV-A (five genomes), and GA2 RSV-A (59 genomes). Recent isolates of the RSV-A clade GA2.1 (a continuation of the GA2 clade) were further divided into three monophyletic groups, with 35 genomes representing the genotype ON1, 22 genomes representing a new group of viruses specific to the study samples from Tennessee that we named genotype TN1, and two genomes with sequences proximal to the divergence point of GA2.1, which we have named genotype TN2 in the context of this study. Both TN1 and TN2 genotypes are supported by Bayesian posterior probabilities of 1 and ML bootstrap values between 99–100%. These findings confirmed co-circulation of multiple RSV clades and genotypes in a given season in the same geographical location.

### Bayesian phylogenetic analyses provide estimates of RSV evolutionary dynamics

**Figure 1:**
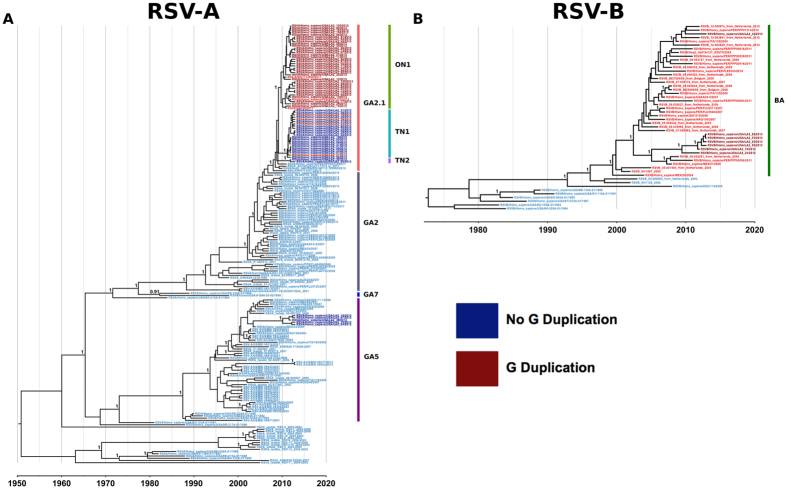
Bayesian maximum clade credibility trees for RSV-A (**A**) and RSV-B (**B**) G gene sequences. Strain names are colored by the presence (red) or absence (blue) of the large G gene duplication, with study samples in darker shades of red and blue. Multiple co-circulating lineages of RSV were observed during the 2012–2013 RSV season. These phylogenies and related analyses suggest that the G gene duplication occurred convergently in two separate genotypes of RSV-A. Bayesian posterior probability >0.9 are provided for key nodes.

**Figure 2:**
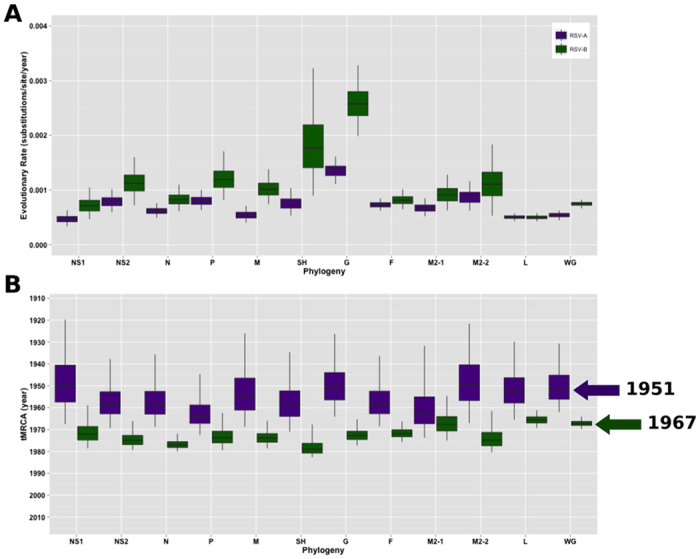
Times to most recent common ancestors (tMCRAs) and mean evolutionary rate estimates inferred by Bayesian analyses. This dataset includes a subset of the available GenBank whole-genome sequences along with the study samples. Estimates are provided for RSV-A (purple) and RSV-B (green) for the whole genome (WG) and each individual gene. (**A**) Evolutionary rates (substitutions/site/year) for RSV-A and RSV-B datasets and (**B**) mean tMRCAs for RSV-A and RSV-B datasets are provided with 95% HPD intervals. The whiskers in each plot extend to the full 95% HPD interval, and the boxes indicate the 25–75% interquartile range of the posterior distribution, thus describing its central tendency. Mean whole-genome tMRCA estimates are indicated with arrows: 1951 for RSV-A and 1967 for RSV-B.

Maximum clade credibility (MCC) trees were constructed using G gene analyses for both RSV-A and RSV-B (Fig. 1). In addition, Bayesian phylogenies using a subset of available GenBank whole genomes, including genomes sequenced by us, were inferred using each individual RSV gene, as well as using the whole genome; these analyses provided substitution rates similar to those reported in previous studies^6^,^9^ for all RSV genes (Fig. 2). In particular, we observed a high substitution rate and a Bayesian highest posterior density (HPD) interval of substitution within the SH gene of RSV-B, similar to previous reports (Table 2)^6^,^9^. The mean estimates of the times to most recent common ancestors (tMRCAs) for the whole-genome dataset suggest that circulating and historical RSV-A lineages share a common ancestor from around 1951 (95% HPD, 1937–1964), and RSV-B likely diverged in 1967 (95% HPD, 1964–1970) based on available whole-genome dataset. Comparing RSV-B G gene phylogenies from our whole-genome dataset to the G gene phylogenies that contain more extensive sampling of all available GenBank full G gene sequences (Fig. S3) indicates that the whole-genome dataset is missing diversity that exists within several RSV-B clades.

Further analysis using Bayesian Tip-association Significance (BaTS) detected that global and local circulation patterns exist. BaTS testing of the RSV-A G gene phylogeny resulted in AI and PS scores of 0.0 and MC scores of 0.009 for the global and local state assignments. The PS and AI scores indicate an overall non-random association of local and global assignments with the phylogenies tested, whereas the MC result indicates the local state is specifically associated with the topologies of the phylogenies tested. Because these results are less than 0.01, they provide strong evidence for these states being topologically associated with the Bayesian phylogenies. No significant correlations were found between RSV disease severity and phylogenetic topologies using BaTS analysis.

### Glycosylation sequon analysis reveals sub-clade specific glycosylation patterns in the G protein

**Figure 3:**
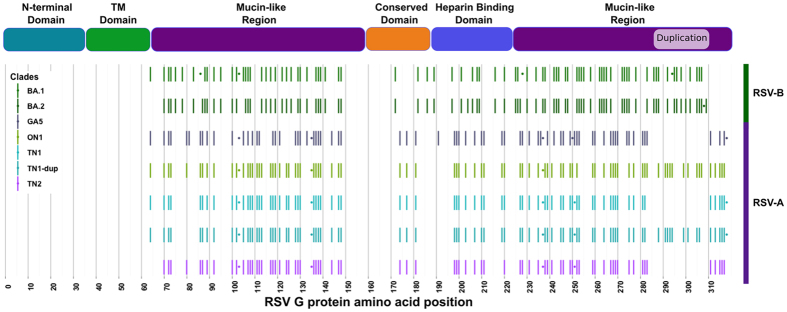
Consensus N- and O-linked glycosylation patterns for the seven study genotypes. The seven genotype-specific consensus glycosylation patterns for O- and N-linked (bars and dots, respectively) glycans are displayed in rows. RSV-A and RSV-B genotypes are indicated with purple and green bars to the right. Each genotype displays a unique glycosylation pattern and duplication status.

Results of NetNGlyc across all study samples show that the N-linked glycosylation sequons in the F gene are relatively conserved. Nearly all of the RSV-A and RSV-B samples have the same N-linked sites (residues 27, 70, 116, 120, and 126) within the F2 domain. However, N-linked glycosylation sequons in the G gene appear to follow a genotype specific pattern, with multiple glycosylation patterns co-circulating simultaneously. Genotype ON1 shows three predicted N-linked glycosylation sites; clades TN1, TN2, and GA5 have five, four, and five sites, respectively (Fig. 3). RSV-B genomes show two different glycosylation patterns. Genotype BA.1 has four glycosylation sites, three of which are consistent with the majority of circulating RSV-B BA.1 genomes. Genotype BA.2 shows a novel RSV-B glycosylation pattern with just one glycosylation site present toward the C-terminal end of the G protein after the G duplication. Similarly, NetOGlyc shows that the O-linked glycosylation patterns for the G protein follow a genotype-specific pattern as well. Seven distinct O-linked patterns are observed in the G protein sequences from our study cohort. Genotype ON1 shows 85 predicted O-linked glycosylation sites, whereas genotype TN1 has 74 and 83 sites (in non-duplicated and duplicated genomes, respectively), genotype TN2 has 74 sites, and genotype GA5 has 72 sites. RSV-B genomes show two different O-linked glycosylation patterns, with 82 sites for genotype BA.1 and 85 sites for genotype BA.2. There were no significant numbers of O-linked glycans predicted for the F protein. Consensus genotype-specific glycosylation patterns were plotted for visual analysis for the G protein (Fig. 3).

### Convergent emergence of a large sequence duplication in the C-terminal region of the G gene

Sequence analysis of the G gene identified seven RSV-B and 39 RSV-A study sample genomes that contained a previously reported insertion within the C-terminal third of the G gene coding sequence^15^,^20^,^22^. The insertion is present as an exact, tandem, in-frame duplication of the same gene region in both the RSV-B and RSV-A genomes, but it is 60 nucleotides in length in RSV-B and 72 nucleotides in RSV-A. Phylogenetic and sequence analyses of the G sequence duplications suggest that the duplication occurred in a convergent fashion, at separate times in both RSV-A GA2.1 genotypes (ON1 and TN1), as well as in the RSV-B group (Fig. S1). All 35 strains from genotype ON1 contained the C-terminal G sequence duplication, while only four out of 22 TN1 genomes contained the G gene sequence duplication; however, none of the TN2 viruses have any sequence duplication. All seven RSV-B genomes contained the G gene sequence duplication, whereas none of the RSV-A GA5 genomes had the duplication.

Global analysis of gene alignments within and between RSV groups showed various deletions and insertions (indels), especially in the G gene, as well as various start- and stop-variant sequences (Table S1). In RSV-A, we observed one indel each in the G and L genes, and two start-site variants in the M2-2 gene. There were also two stop-site variants in the G gene dataset. Within the RSV-B dataset we observed four indels in the G gene with three stop-site variants. We also observed two start-site variants in the M2-2 gene. Comparing RSV-A to RSV-B, we observed an additional seven indels; two in the G gene, four in the L gene, and one in the SH gene. In addition to these intergroup indels, there were two variant stop sites found in M2-1 between groups. Interestingly, one M2-2 start-site variant was shared between subtypes while one each was unique in RSV-A and RSV-B leading to only three start-site variants observed in the intergroup comparison.

**Figure 4:**
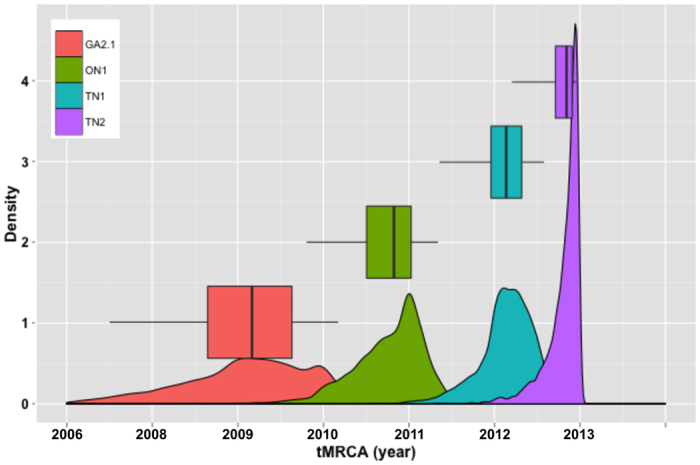
Divergence time estimates from a Bayesian divergence dating analysis of the RSV-A G gene sequences. The GA2.1 clade consists of ON1 genotypes containing only sequences with the G gene duplication, TN1 genotypes containing sequences with mostly non-duplicated G genes and four interleaved G gene duplication sequences, and TN2 genotypes containing only sequences lacking the G gene duplication. Divergence estimates suggest clade GA2.1 originated from a non-duplicated ancestor, with the duplication being convergently gained first in genotypes ON1 and then in TN1. This hypothesis of convergent G gene duplications is supported by divergence estimates that largely do not overlap between genotypes ON1 and TN1. The whiskers in each plot extend to the full 95% HPD interval, and the boxes indicate the 25–75% interquartile range of the posterior distribution, thus describing its central tendency.

The Bayesian analysis of the G gene of RSV-A also supported the hypothesis that the G gene duplication occurred at least twice in a convergent manner within the RSV-A genotypes ON1 and TN1 (Fig. 1A). This is evident from the interleaving of the RSV-A genomes containing the C-terminal G sequence duplication with non-duplicated genomes within the G gene phylogeny, as well as by the divergence dating estimates for the recent GA2.1 strains and the contained genotypes: ON1, TN1, and TN2 (Fig. 4). These results suggest that genotype ON1 diverged first in late 2009 (95% HPD, 2009.0–2010.4), followed by genotype TN1 in early 2011 (95% HPD, 2010.4–2011.6) (a local Tennessee clade), and finally by genotype TN2 in late 2011 (95% HPD, 2011.4–2012.0). Because the latter two genotypes appear to have evolved from a non-duplicated ancestral G gene sequence, genotype TN1 most likely acquired the duplication convergently. This hypothesis is also supported by the minimal overlap in the 95% HPD intervals of divergence time estimates for genotypes ON1 and TN1. Additionally, a Bayesian analysis was performed using BEAST with duplication/no duplication as a trait to provide added support for the convergence hypothesis. The results exclude the possibility of trait reversion and confirm that the duplication evolved convergently in these two genotypes (Fig. S4).

Further, we performed whole-genome phylogenetic analysis using 21 RSV genome sequences from the 2013–2014 season along with the 2012–2013 dataset. Although relatively few genomes were available, the resulting maximum likelihood phylogeny (Fig. S5) suggests a switch from RSV-A to RSV-B predominance. However, we also noted the continued circulation of the RSV-A ON1 genotype viruses, which all contain the G gene sequence duplication, suggesting the G gene sequence duplication is potentially moving toward fixation, possibly due to a fitness advantage over the non-duplicated RSV-A genomes.

## Discussion

Here, we have identified multiple co-circulating RSV clades and sub-clades infecting infants within the central Tennessee region during the 2012–2013 season, where substantial RSV genetic diversity was observed both between and within the RSV-A and -B groups. This diversity was especially evident within the G gene, although additional sequence variation was present in the F and select regions of the L gene. We observed seven distinct G gene variants in our dataset, as defined by both G gene duplication and glycosylation status. This observed diversity is possibly a result of RSV evolution to evade host adaptive immune responses^26^,^27^,^28^.

Our findings demonstrate that during the 2012–2013 RSV season, three distinct lineages of RSV were co-circulating within the central Tennessee region. This supports previous reports of multiple RSV types co-circulating in a given season^13^,^16^,^29^. Furthermore, RSV-A clade GA2.1 appears to have predominated 83.1% of observed infections in our study cohort. A local subgroup of RSV-A within clade GA2.1 was observed circulating only in central Tennessee (genotype TN1). The proposed genotypes TN1 and TN2 appear to be novel additions to the diversity of RSV-A. This is supported by the high posterior probabilities and bootstrap values on the branches leading to these groups of viruses from both the Bayesian and ML trees respectively. Furthermore the divergence times for TN1 and TN2 appear to be after that of ON1, leading us to conclude that they are not merely representative of the NA1 genotype. Complete NA1 genomes were not available and thus not included for analysis in this study.

Here we observed tree topologies with little or no temporal and or geographic patterns and others with strong geographic and temporal patterns. Over the long-term, most genetically diverse strains, both RSV-A and RSV-B, circulate globally over a relatively short time period. In our study and other studies, such as *Agoti et al.*^9^, localized strain evolution is sometimes apparent within this global strain circulation. Genotype TN1 appears to be localized to central Tennessee RSV, while several RSV-B strains were noted to be local to Kilifi, Kenya in the Agoti study^9^. We tested the assumption of localized clades using BaTS with a significant result, suggesting that genotype TN1 and Tennessee GA5 viruses were being locally transmitted during the 2012–2013 season. With broader genomic surveillance of RSV, these epidemiological patterns can be studied more closely and the origins of various lineages could be determined.

Comparing publicly available whole-genome RSV sequences to all available full-length G gene sequences indicated that, while whole RSV-A genomes are largely representative of known RSV-A diversity, the corresponding RSV-B whole-genome dataset is missing diversity within the RSV-B BA clade, which may explain the relatively large ranges obtained for the Bayesian substitution rate estimates for many of the genes compared with those for RSV-A. With the addition of seven new RSV-B whole-genomes, our tMRCA estimate for RSV-B is improved over previous estimates^6^; however, the estimate would likely be improved with additional whole-genome sequences of historical RSV-B genomes.

In general, the glycosylation and indel patterns in the RSV-B dataset appear more varied, which supports the idea that RSV-B seems to have more G gene plasticity than RSV-A. The differences in G glycosylation in both the RSV-A and the RSV-B groups may help the virus spread and overcome population immunity. The F protein has a conserved glycosylation pattern across RSV-A and RSV-B viruses and appears to only permit N-linked glycans in the F2 domain. The high degree of conservation in the F1 domain is likely needed to maintain fitness as it is required to retain a functional fusion mechanism, as this protein undergoes a complex conformational change once attachment triggers fusion^30^. The overall conservation of F juxtaposed against the variability in G suggests F to be a more suitable target for universal therapeutics and vaccines^31^.

The convergent appearances of C-terminal G gene sequence duplications in the same location for multiple RSV lineages suggest that the G protein plasticity for tolerance of insertions, and potentially indicate a mechanism for the development of novel immune evasion strategies. We observed large 72 nt duplication in the G protein of RSV-A with two stop-variants, and four indels of various sizes with three stop-variants in the RSV-B G protein dataset. The two major duplications likely also indicate that the duplications impart some level of selective advantage for the virus as the duplication appears to have reached fixation in RSV-B genomes and may be moving toward fixation in RSV-A, although improved RSV surveillance/sampling is required to know this for sure. A recent study by *Hotard et al.* showed an association between the 60 nt G C-terminal sequence duplication in RSV-B and an enhancement of the attachment function of the G protein^32^. It is possible the 72 nt duplication in RSV-A similarly enhances the G protein, thus providing a selective advantage to duplicated viruses. As previously reported, there may be a mutation in the G gene that primes duplication to occur, making it more likely to happen in a convergent manner. It has been proposed that stem loop structures form in the replicating RNA strand causing the polymerase to pause and reinitiate replication further back on the template^15^. The apparent observation of this duplication occurring repeatedly in RSV-A of the same length and location supports this proposed mechanism as an explanation for duplication events.

Comparison of RSV-A and RSV-B whole-genome sequences shows that RSV-B contains more indels within the G gene, suggesting that different selection pressures exist between these groups. Overall higher substitution rates in RSV-B and specifically the difference in the SH gene substitution rates between RSV-B and RSV-A further support this, although this could also be a result of poor RSV-B surveillance. This apparent poor surveillance supports the need to adopt a whole-genome sequencing approach for future RSV studies. The observation that the local Tennessee genotype, TN1, did not reach global circulation supports this notion, although poor global surveillance of RSV is an alternative explanation. Similarly, at least one RSV-B duplicated genome exists in our dataset from 1996, earlier than the previous first observation in 1999 of 60 nt duplication in the G gene of RSV-B^21^. It should be noted that the 1996 genome was located in a separate clade from the BA clade where RSV-B duplicated genomes originate, supporting convergence as a mechanism for increased probability of success of these mutations reaching global circulation.

One major limitation of this study was the lack of extensive historical and contemporary sampling to place our whole-genome sequences in context with. For instance, without a robust surveillance network for RSV, it is hard to know for sure if the TN1 genotype was truly geographically isolated to Tennessee during the 2012–2013 season. Another notable limitation is the relatively small sample sizes and lower success rate of whole genome sequence due to RNA degradation, to perform statistical associations of clinical and genetic data. Both point toward the need for expanded surveillance and coordination of clinical data collection.

## Methods

### Study population

INSPIRE is an observational, population-based, longitudinal study of previously healthy, term infants enrolled near birth. Eligible infants were born between June and December and were on average ≤6 months of age during sampling for this study. Infants met our case definition of a respiratory illness visit based on responses to the bi-weekly respiratory illness surveillance questionnaires: a parent indicates ONE of the following major symptoms or diagnoses: wheezing, difficulty breathing, or told that your baby had a positive RSV test OR ANY TWO of the following minor symptoms or diagnoses: fever, runny nose/congestion/snotty nose, cough, ear infection (otitis media), or hoarse cry. Onset of symptoms must have been in the prior 7 days. If the infant met pre-specified criteria for a respiratory illness visit, an in-person visit was performed within 7 days of the report of symptom onset. Based on a Bi-weekly viral surveillance conducted during November through March, enrolled infants meeting pre-specified criteria for a respiratory illness undergo an in-person visit and nasal wash sampling for viral determination using PCR. Informed consent was obtained from the legal guardians of each infant. All procedures were approved by the ethical committee of the Vanderbilt University Institutional Review Board and were carried out in “accordance” with the approved guidelines. Demographic data – including age, sex, race and ethnicity – were recorded at the time of enrollment. Singleplex qRT-PCR assays for multiple viruses, including, RSV, human rhinovirus, human enterovirus, and human ribonuclease P (RNAseP) in nasal washes were performed according to standardized protocols^25^,^33^,^34^, and are described previously^25^. The algorithm used to select samples included all samples with a doctor diagnosis of bronchiolitis, plus, illnesses from the lower end of the severity spectrum based on an ordinal severity score^35^ with an RSV cycle threshold greater than 20. This resulted in samples spanning winter virus season with variable severity. Samples from individuals with multiple RSV nasal washes were also included.

### RNA extraction and RT-PCR

Extraction of the viral RNA was performed at the J. Craig Venter Institute (JCVI) in Rockville, MD with 140 μl of the nasal wash sample using the ZR 96 Viral RNA kit (Zymo Research Corporation, Irvine, CA, USA). Four forward reverse transcription (RT) primers were designed and four sets of PCR primers were manually picked from primers designed across a consensus of complete RSV genome sequences using JCVI’s automated primer design tool^36^. The four forward RT primers were diluted to 2 μM and pooled in equal volumes. cDNA was generated from 4 μl undiluted RNA, using the pooled forward primers and SuperScript III Reverse Transcriptase (Thermo Fisher Scientific, Waltham, MA, USA). Four independent PCR reactions were performed on 2 μl of cDNA template using either AccuPrime Taq DNA Polymerase (Thermo Fisher Scientific) or Phusion High Fidelity DNA Polymerase (New England Biolabs, Ipswich, MA, USA) to generate four overlapping ~4-kb amplicons across the genome. Amplicons were verified on 1% agarose gels, and excess primers and dNTPs were removed by treatment with Exonuclease I (New England Biolabs) and shrimp alkaline phosphatase (Affymetrix, Santa Clara, CA, USA) for 37 °C for 60 min, followed by incubation at 72 °C for 15 min. Amplicons were quantitated using a SYBR Green dsDNA detection assay (SYBR Green I Nucleic Acid Gel Stain, Thermo Fisher Scientific), and all four amplicons per genome were pooled in equal concentration.

### RSV whole-genome sequencing

For samples sequenced using the Ion Torrent PGM (Thermo Fisher Scientific), 100 ng of pooled DNA amplicons were sheared for 7 min, and Ion-Torrent-compatible barcoded adapters were ligated to the sheared DNA using the Ion Xpress Plus Fragment Library Kit (Thermo Fisher Scientific) to create 400-bp libraries. Libraries were pooled in equal volumes and cleaned with Ampure XP reagent (Beckman Coulter, Inc., Brea, CA, USA). Quantitative PCR was performed on the pooled, barcoded libraries to assess the quality of the pool and to determine the template dilution factor for emulsion PCR. The pool was diluted appropriately and amplified on Ion Sphere Particles (ISPs) during emulsion PCR on the Ion One Touch 2 instrument (Thermo Fisher Scientific). The emulsion was broken, and the pool was cleaned and enriched for template-positive ISPs on the Ion One Touch ES instrument (Thermo Fisher Scientific). Sequencing was performed on the Ion Torrent PGM using 316v2 or 318v2 chips (Thermo Fisher Scientific).

For samples requiring extra coverage, in addition to Ion Torrent sequencing, Illumina libraries were prepared using the Nextera DNA Sample Preparation Kit (Illumina, Inc., San Diego, CA, USA) with half reaction volumes. Briefly, 25 ng of pooled DNA amplicons were tagmented at 55 °C for 5 min. Tagmented DNA was cleaned with the ZR-96 DNA Clean & Concentrator Kit (Zymo Research Corporation) and eluted in 25 μl resuspension buffer. Illumina sequencing adapters and barcodes were added to tagmented DNA via PCR amplification, where 20 μl tagmented DNA was combined with 7.5 μl Nextera PCR Master Mix, 2.5 μl Nextera PCR Primer Cocktail and 2.5 μl of each index primer (Integrated DNA Technologies, Coralville, IA, USA) for a total volume of 35 μl per reaction. Thermocycling was performed with 5 cycles of PCR, as per the Nextera DNA Sample Preparation Kit protocol (3 min at 72 °C, denaturation for 10 sec at 98 °C, annealing for 30 sec at 63 °C and extension for 3 min at 72 °C) to create a dual-indexed library for each sample. After PCR amplification, 10 μl of each library was pooled into a 1.5-mL tube, and the pool was cleaned two times with Ampure XP reagent (Beckman Coulter, Inc.) to remove all leftover primers and small DNA fragments. The first cleaning used a 1.2× volume of the Ampure reagent, while the second cleaning used a 0.6× volume of the Ampure reagent. The cleaned pool was sequenced on the Illumina MiSeq v2 instrument (Illumina, Inc.) with 300-bp paired-end reads.

### RSV genome assembly and annotation

Sequence reads were sorted by barcode, trimmed, and de novo assembled using CLC Bio’s *clc_novo_assemble* program^37^, and the resulting contigs were searched against custom, full-length RSV nucleotide databases to find the closest reference sequence. All sequence reads were then mapped to the selected reference RSV sequence using CLC Bio’s *clc_ref_assemble_long* program^38^. At loci where both Ion Torrent and Illumina sequence data agreed on a variation (compared with the reference sequence), the reference sequence was updated to reflect the difference. A final mapping of all next-generation sequences to the updated reference sequences was performed with CLC Bio’s *clc_ref_assemble_long* program^38^. Curated assemblies were validated and annotated with the viral annotation software called Viral Genome ORF Reader, VIGOR 3.0^39^, before submission to GenBank. VIGOR was used to predict genes, perform alignments, ensure the fidelity of open reading frames, correlate nucleotide polymorphisms with amino acid changes, and detect any potential sequencing errors. The annotation was subjected to manual inspection and quality control before submission to GenBank. All sequences generated as part of this study were submitted to GenBank as part of the Bioproject ID PRJNA225816.

### Phylogenetic analyses

#### Sequence collection

All available full-length human RSV-A and RSV-B genomes were downloaded from GenBank on June 24 2014. Any viral isolates that contained “mutant” or other key words indicating *in vitro* modifications were removed from the dataset after an initial ML phylogenetic analysis. The remaining public genomes were then combined with the 71 RSV genomes from study samples collected during the 2012–2013 winter RSV season. Full genomes were then annotated using VIGOR 3.0^39^ to ensure consistent gene annotations across all genomes. Each of the eleven RSV genes were separated into gene-specific fasta files for gene-based phylogenetic analyses. Genomes without complete gene counts or containing partial gene annotations after processing with VIGOR were excluded from further analysis.

#### Maximum likelihood analyses of whole-genome and G-gene-specific RSV sequences

The nucleotide substitution model used for all phylogenetic analyses was a general time reversible model with a nucleotide site-specific rate heterogeneity with four rate categories and invariant sites (GTR-IG), as determined by jModelTest2.4^40^. MAFFT^41^ was used to create whole-genome and G-gene-specific alignments. All alignments were checked and edited as appropriate. Maximum likelihood phylogenies were inferred using an adaptive best tree search on the GARLI Web Service 2.0^42^ to statistically ensure the best tree, as measured by log likelihood scores, estimated over over 1000 bootstrap replicates. All branches leading to study sequences were supported by bootstap value >70. Clade designations for the study sequences were determined by examining bootstrap support on these branches using the reduced G gene phylogeny (S2). The resultant tree was labeled with viral strain names and colored using in-house PERL scripts.

#### Bayesian phylogenetic analyses of RSV-A and RSV-B genomes

The whole-genome maximum likelihood tree was used as a guide to select a subset of viral genomes for Bayesian phylogenetic analyses, including genomes with unique phylogenetic histories and commonly used reference genomes. To determine if our data exhibited temporal qualities, we performed an exploratory analysis with Path-O-Gen (available at http://tree.bio.ed.ac.uk/software/pathogen/). Neighbor joining trees generated with RSV-A-only and RSV-B-only genomes were used to measure root-to-tip divergence using Path-O-Gen, which showed that both RSV datasets contained enough temporal signal to proceed with time-based Bayesian analyses. All Bayesian analyses were performed using BEAST v1.8^43^,^44^ on the CIPRES Science Gateway^45^. Whole-genome and gene-specific phylogenies were inferred using Markov chain Monte Carlo sampling chains of 100 million to one billion in length, with parameters and trees recorded to ensure 10,000 samples per run. The GTR-IG substitution model was used and tip dating with precision to the sampling year was employed for all trees. All genes were analyzed using a lognormal relaxed clock. Default priors were used for each analysis except for the ucld.mean procedure for which we used the CTMC rate reference prior^46^. For the G gene analysis, we performed divergence dating on RSV-A by constraining four clades and genotypes most closely related to two separate lineages of viruses with G duplications present. We also performed a trait analysis with BEAST using duplication/no duplication as leaf states to reconstruct the evolutionary history of the duplication trait. All analyses were evaluated with Tracer v1.6 (available at http://tree.bio.ed.ac.uk/software/tracer/) to determine the success of the chain sampling based on effective sample size values for each parameter. Additional chains were run as needed. For each analysis, we constructed a maximum clade credibility tree using TreeAnnotator v1.8.1, available for download with BEAST.

### Glycosylation Prediction

Two surface glycoproteins, F and G, were analyzed using NetNGlyc^47^ and NetOGlyc^48^ software. Multifasta files were loaded into the web interface and output was saved, and then parsed with custom PERL scripts to produce a spreadsheet of glycosylation sequons and amino acid coordinates for the N-linked and O-linked glycans. The coordinates were used to to produce visualizations in R using the ggplot2 package^49^ of the overall consensus glycosylation patterns on the G protein for the various clades and genotypes identified in this study.

### BaTS analysis for detecting global versus local circulations patterns with tree topologies

The Bayesian RSV-A G gene phylogeny dataset was used to analyze signals of global versus local circulation within our dataset. Global versus local circulation status was assigned to each G gene sequence. BaTS analysis^50^ was performed using a total of 9001 Bayesian phylogenies of RSV-A G gene sequences from the previous analysis. The BaTS analysis was run in single mode with 100 replicates using two states (global and local). BaTS results are reported with three scores: AI, PS, and MC. The AI and PS scores are two methods for testing the overall structure of all traits tested to tree topologies, whereas the MC scores indicate the association of specific traits with the tree topologies. Any score less than 0.05 indicates an association for that particular test and scores less than 0.01 are indicative of strong associations.

## Additional Information

**How to cite this article**: Schobel, S. A. *et al*. Respiratory Syncytial Virus whole-genome sequencing identifies convergent evolution of sequence duplication in the C-terminus of the G gene. *Sci. Rep.* **6**, 26311; doi: 10.1038/srep26311 (2016).

## Supplementary Material

Supplementary Table S1

Supplementary Fig S1

Supplementary Fig S2

Supplementary Fig S3

Supplementary Fig S4

Supplementary Fig S5

Supplementary Fig S6

## Figures and Tables

**Table 1:** Demographics and clinical characteristics of enrolled infants included (n = 99) and excluded (n = 99) in this study during the 2012–2013 season.

**Demographics and Clinical Characteristics**	**Included Infants with RSV ARTIs (n** = 99)*^†^ **[106 total samples]**	**Excluded Infants with RSV ARTIs (n** = **99) [103 total samples]**
9Age (weeks)	22 (13–27)	21 (13–27)
Female gender	42 (42.42%)	48 (48.48%)
Race		
Black	15 (15.15%)	7 (7.07%)
White	72 (72.73%)	76 (76.77%)
Other§	12 (12.12%)	6 (6.06%)
Hispanic ethnicity	11 (11.11%)	10 (10.10%)
Gestational age (weeks)	39 (39–40)	39 (39–40)

Twelve infants had multiple RSV ARTIs over the surveillance period; three of these infants had two RSV ARTIs and only the earliest collected sample was subjected to whole genome RSV sequencing.

ARTIs = acute respiratory tract infections; RSV = Respiratory Syncytial Virus.

^*^Data are presented as the number (%) for categorical variables or median (interquartile range) for continuous variables.

^†^Percentage calculated for children with complete data.

^§^Category includes subjects of mixed race.

**Table 2:** Mean evolutionary rates (substitutions/site/year) and times to most recent common ancestors (tMRCAs) as inferred by Bayesian analysis.

	**tMRCA (95% HPD)**	**MeanRate (95% HPD)**
RSV-A WG	1951 (1937–1964)	5.68 × 10^−4^ (6.55 × 10^−4^ to 4.87 × 10^−4^)
RSV-B WG	1967 (1964–1970)	7.47 × 10^−4^ (8.22 × 10^−4^ to 6.64 × 10^−4^)
RSV-A G	1949 (1928–1966)	1.35 × 10^−3^ (1.60 × 10^−3^ to 1.10 × 10^−3^)
RSV-B G	1972 (1966–1978)	2.59 × 10^−3^ (3.28 × 10^−3^ to 1.98 × 10^−3^)

WG = whole genome; G = G gene; HPD = highest posterior density.
