# Supplementary material for: Respiratory Syncytial Virus whole-genome sequencing identifies convergent evolution of sequence duplication in the C-terminus of the G gene
Source: Sci Rep. 2016 May 23;6:26311. doi: 10.1038/srep26311 (PMC4876326; doi:10.1038/srep26311)
Supplement: Supplementary Table S1 [file srep26311-s1.pdf]

Title: Respiratory Syncytial Virus whole-genome sequencing identifies convergent evolution of sequence duplication in the C-terminus of the G gene.

Seth A. Schobel[1,2,7], Karla M. Stucker[1], Martin L. Moore[3], Larry J. Anderson[3], Emma K. Larkin[5,6], Jyoti Shankar[1], Jayati Bera[1], Vinita Puri[1], Meghan H. Shilts[1], Christian Rosas-Salazar[4], Rebecca A. Halpin[1], Nadia Fedorova[1], Susmita Shrivastava[2], Timothy B. Stockwell[2], R. Stokes Peebles[5,6], Tina V. Hartert[5,6], Suman R. Das[1*]

[1]Infectious Diseases and [2]Bioinformatics Group, J. Craig Venter Institute, Rockville, MD

[3]Division of Infectious Diseases, Department of Pediatrics, Emory University School of Medicine and Children's Healthcare of Atlanta, Atlanta, GA

[4]Division of Allergy, Immunology, and Pulmonary Medicine, Department of Pediatrics, Vanderbilt University School of Medicine, Nashville, TN

[5]Department of Medicine, Vanderbilt University School of Medicine, Nashville, TN

[6]Division of Allergy, Pulmonary, and Critical Care Medicine, Department of Medicine, Vanderbilt University School of Medicine, Nashville, TN

[7]Center for Bioinformatics and Computational Biology, University of Maryland, College Park, MD

Key Words: Respiratory Syncytial Virus, next-generation sequencing, evolution, disease severity

Journal: Scientific Reports

[*]**Corresponding author:**

Suman Ranjan Das

Infectious Disease Group

J. Craig Venter Institute

Rockville, Maryland 20850

Phone: 301-795-7328

Fax: 301-795-7070

E-mail: sdas@jcvi.org

Table S1. Observed indels and start- and stop-site variants within RSV-A, within RSV-B, and between the RSV-A and RSV-B groups. More indels are observed within the RSV-B group, particularly in the G gene, suggesting greater plasticity of G in RSV-B. Additionally, RSV-A and RSV-B differences, especially in L, suggest the potential for functional differences in their polymerases that may lead to this apparent greater rate of indels in RSV-B.

Group	Gene	Indel
RSV-A	F	none
	G	**780-852**, 966-stop, 969-stop
	L	400-406
	M	none
	M2-1	none
	M2-2	1-start, 7-start
	N	none
	NS1	none
	NS2	none
	P	none
	SH	none
RSV-B	F	none
	G	471-477, 673-685, 704-707, **793-853**, 954-stop, 963-stop, 975-stop
	L	none
	M	none
	M2-1	none
	M2-2	1-start, 10-start
	N	none
	NS1	none
	NS2	none
	P	none
	SH	none
Inter Group	F	none
	G	471-477, **634-637**, 673-685, 704-707, **793-853**, **857-929**, **1003-1008**, 1029-stop, 1038-stop, 1042-stop, 1045-stop, 1050-stop
	L	400-406, **5193-5196**, **5280-5283**, **6504-stop**, **6507-stop**
	M	none
	M2-1	**582-stop**, **585-stop**
	M2-2	1-start, 10-start, 16-start
	N	none
	NS1	none
	NS2	none
	P	none
	SH	**180-183**

Supplemental Files
