# Supplementary material for: Respiratory Syncytial Virus whole-genome sequencing identifies convergent evolution of sequence duplication in the C-terminus of the G gene
Source: Sci Rep. 2016 May 23;6:26311. doi: 10.1038/srep26311 (PMC4876326; doi:10.1038/srep26311)
Supplement: Supplementary Fig S1 [file srep26311-s2.pdf]

Title: Respiratory Syncytial Virus whole-genome sequencing identifies convergent evolution of sequence duplication in the C-terminus of the G gene.

Seth A. Schobel[1,2,7], Karla M. Stucker[1], Martin L. Moore[3], Larry J. Anderson[3], Emma K. Larkin[5,6], Jyoti Shankar[1], Jayati Bera[1], Vinita Puri[1], Meghan H. Shilts[1], Christian Rosas-Salazar[4], Rebecca A. Halpin[1], Nadia Fedorova[1], Susmita Shrivastava[2], Timothy B. Stockwell[2], R. Stokes Peebles[5,6], Tina V. Hartert[5,6], Suman R. Das[1*]

[1]Infectious Diseases and [2]Bioinformatics Group, J. Craig Venter Institute, Rockville, MD

[3]Division of Infectious Diseases, Department of Pediatrics, Emory University School of Medicine and Children's Healthcare of Atlanta, Atlanta, GA

[4]Division of Allergy, Immunology, and Pulmonary Medicine, Department of Pediatrics, Vanderbilt University School of Medicine, Nashville, TN

[5]Department of Medicine, Vanderbilt University School of Medicine, Nashville, TN

[6]Division of Allergy, Pulmonary, and Critical Care Medicine, Department of Medicine, Vanderbilt University School of Medicine, Nashville, TN

[7]Center for Bioinformatics and Computational Biology, University of Maryland, College Park, MD

Key Words: Respiratory Syncytial Virus, next-generation sequencing, evolution, disease severity

Journal: Scientific Reports

[*]**Corresponding author:**

Suman Ranjan Das

Infectious Disease Group

J. Craig Venter Institute

Rockville, Maryland 20850

Phone: 301-795-7328

Fax: 301-795-7070

E-mail: sdas@jcvi.org

SUPPLEMENTAL FIGURE LEGEND

Figure S1. Maximum likelihood phylogeny of 545 RSV whole-genome sequences, including 474 downloaded from GenBank on June 24, 2014 and 71 study sequences. Whole-genomes sequences include mutant and lab-constructed strains. RSV-A study isolates are depicted in purple, while RSV-B study isolates are depicted in green. Co-circulating strains from multiple clades were present during the 2012-2013 RSV season in central Tennessee. Bootstrap values > 70% for key nodes relevant to this study are provided for over 1,000 replicates, and the scale bar indicates nucleotide substitutions per site per year.

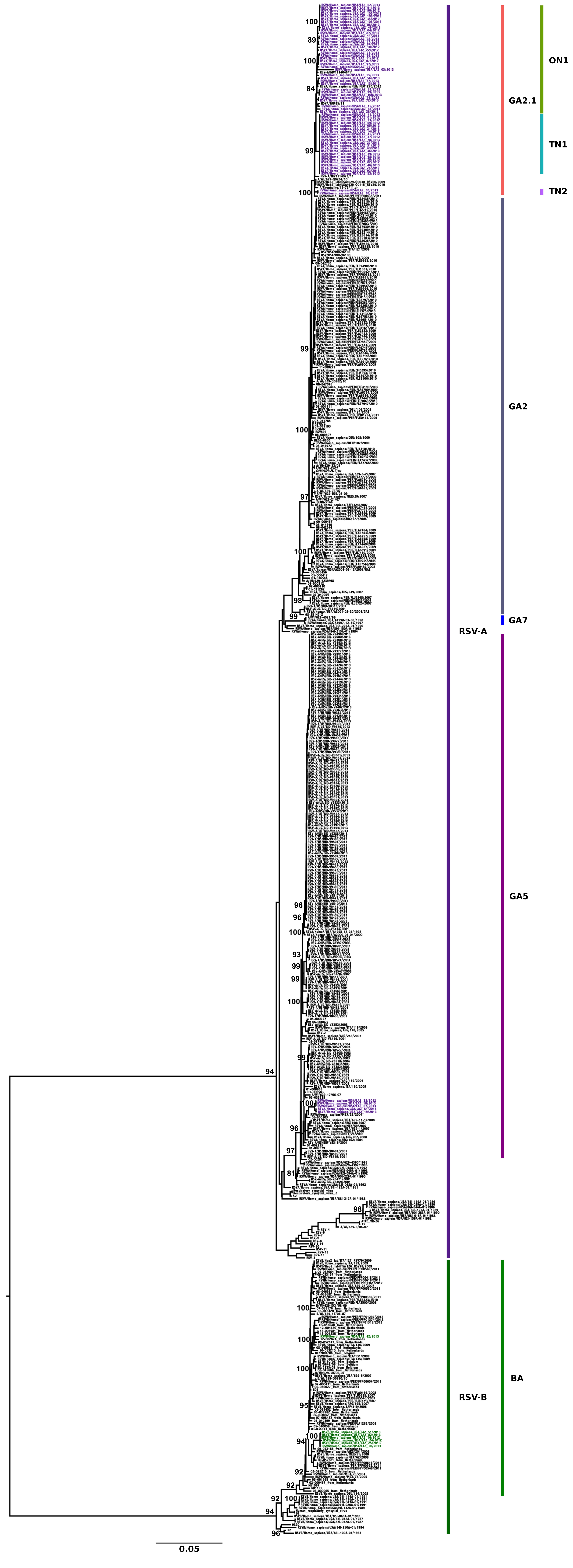
ON1
GA2.1
TN1
TN2
GA2
GA7
RSV-A
GA5
BA
RSV-B
0.05
