# Supplementary material for: Respiratory Syncytial Virus whole-genome sequencing identifies convergent evolution of sequence duplication in the C-terminus of the G gene
Source: Sci Rep. 2016 May 23;6:26311. doi: 10.1038/srep26311 (PMC4876326; doi:10.1038/srep26311)
Supplement: Supplementary Fig S4 [file srep26311-s5.pdf]

Title: Respiratory Syncytial Virus whole-genome sequencing identifies convergent evolution of sequence duplication in the C-terminus of the G gene.

Seth A. Schobel[1,2,7], Karla M. Stucker[1], Martin L. Moore[3], Larry J. Anderson[3], Emma K. Larkin[5,6], Jyoti Shankar[1], Jayati Bera[1], Vinita Puri[1], Meghan H. Shilts[1], Christian Rosas-Salazar[4], Rebecca A. Halpin[1], Nadia Fedorova[1], Susmita Shrivastava[2], Timothy B. Stockwell[2], R. Stokes Peebles[5,6], Tina V. Hartert[5,6], Suman R. Das[1*]

[1]Infectious Diseases and [2]Bioinformatics Group, J. Craig Venter Institute, Rockville, MD

[3]Division of Infectious Diseases, Department of Pediatrics, Emory University School of Medicine and Children's Healthcare of Atlanta, Atlanta, GA

[4]Division of Allergy, Immunology, and Pulmonary Medicine, Department of Pediatrics, Vanderbilt University School of Medicine, Nashville, TN

[5]Department of Medicine, Vanderbilt University School of Medicine, Nashville, TN

[6]Division of Allergy, Pulmonary, and Critical Care Medicine, Department of Medicine, Vanderbilt University School of Medicine, Nashville, TN

[7]Center for Bioinformatics and Computational Biology, University of Maryland, College Park, MD

Key Words: Respiratory Syncytial Virus, next-generation sequencing, evolution, disease severity

Journal: Scientific Reports

[*]**Corresponding author:**

Suman Ranjan Das

Infectious Disease Group

J. Craig Venter Institute

Rockville, Maryland 20850

Phone: 301-795-7328

Fax: 301-795-7070

E-mail: sdas@jcvi.org

SUPPLEMENTAL FIGURE LEGEND

Figure S4. Bayesian maximum clade credibility tree showing trait analysis of the duplication versus no duplication in RSV-A G gene sequences. The phylogeny shows the duplicated G state (red) to have evolved independently with no state reversions in the ON1 and TN1 genotypes. These results confirm the convergence hypothesis as an explanation for repeated acquisitions of the G gene duplication in disparate genotypes. Bayesian posterior probability > 0.9 are provided for key nodes.

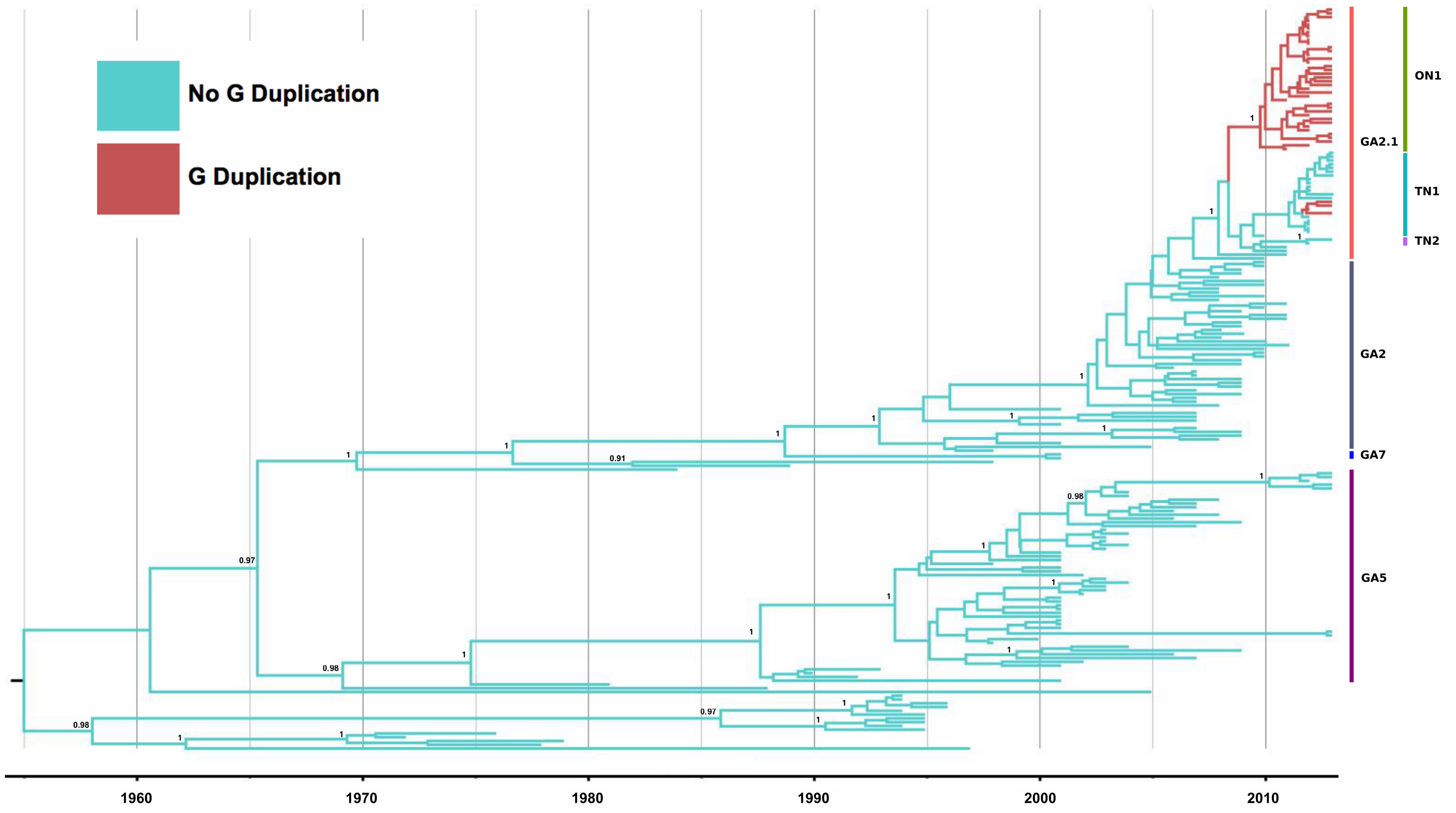

No G Duplication
G Duplication
GA2.1
ON1
TN1
TN2
GA2
GA7
GA5
1960
1970
1980
1990
2000
2010
