# Supplementary material for: Respiratory Syncytial Virus whole-genome sequencing identifies convergent evolution of sequence duplication in the C-terminus of the G gene
Source: Sci Rep. 2016 May 23;6:26311. doi: 10.1038/srep26311 (PMC4876326; doi:10.1038/srep26311)
Supplement: Supplementary Fig S5 [file srep26311-s6.pdf]

Title: Respiratory Syncytial Virus whole-genome sequencing identifies convergent evolution of sequence duplication in the C-terminus of the G gene.

Seth A. Schobel[1,2,7], Karla M. Stucker[1], Martin L. Moore[3], Larry J. Anderson[3], Emma K. Larkin[5,6], Jyoti Shankar[1], Jayati Bera[1], Vinita Puri[1], Meghan H. Shilts[1], Christian Rosas-Salazar[4], Rebecca A. Halpin[1], Nadia Fedorova[1], Susmita Shrivastava[2], Timothy B. Stockwell[2], R. Stokes Peebles[5,6], Tina V. Hartert[5,6], Suman R. Das[1*]

[1]Infectious Diseases and [2]Bioinformatics Group, J. Craig Venter Institute, Rockville, MD

[3]Division of Infectious Diseases, Department of Pediatrics, Emory University School of Medicine and Children's Healthcare of Atlanta, Atlanta, GA

[4]Division of Allergy, Immunology, and Pulmonary Medicine, Department of Pediatrics, Vanderbilt University School of Medicine, Nashville, TN

[5]Department of Medicine, Vanderbilt University School of Medicine, Nashville, TN

[6]Division of Allergy, Pulmonary, and Critical Care Medicine, Department of Medicine, Vanderbilt University School of Medicine, Nashville, TN

[7]Center for Bioinformatics and Computational Biology, University of Maryland, College Park, MD

Key Words: Respiratory Syncytial Virus, next-generation sequencing, evolution, disease severity

Journal: Scientific Reports

[*]**Corresponding author:**

Suman Ranjan Das

Infectious Disease Group

J. Craig Venter Institute

Rockville, Maryland 20850

Phone: 301-795-7328

Fax: 301-795-7070

E-mail: sdas@jcvi.org

SUPPLEMENTAL FIGURE LEGEND

Figure S5. Maximum likelihood phylogeny of a pruned RSV whole-genome dataset. The dataset for this phylogeny is the same as for Figure S2, with the addition of 20 genomes collected during the INSPIRE study in Tennessee during the 2013-2014 RSV season. RSV-A study isolate names are depicted in purple, while RSV-B study isolate names are depicted in green; the 2013-2014 isolate names are depicted in orange. The 2013-2014 season in Tennessee witnessed a switch to RSV-B dominance, as well as the continued circulation of RSV-A genomes containing the G gene duplication (6 out of 6 RSV-A 2013-2014 study strains). Bootstrap support > 70% from 1,000 replicates was included for nodes that were important for this study. The scale bar indicates nucleotide substitutions per site per year.

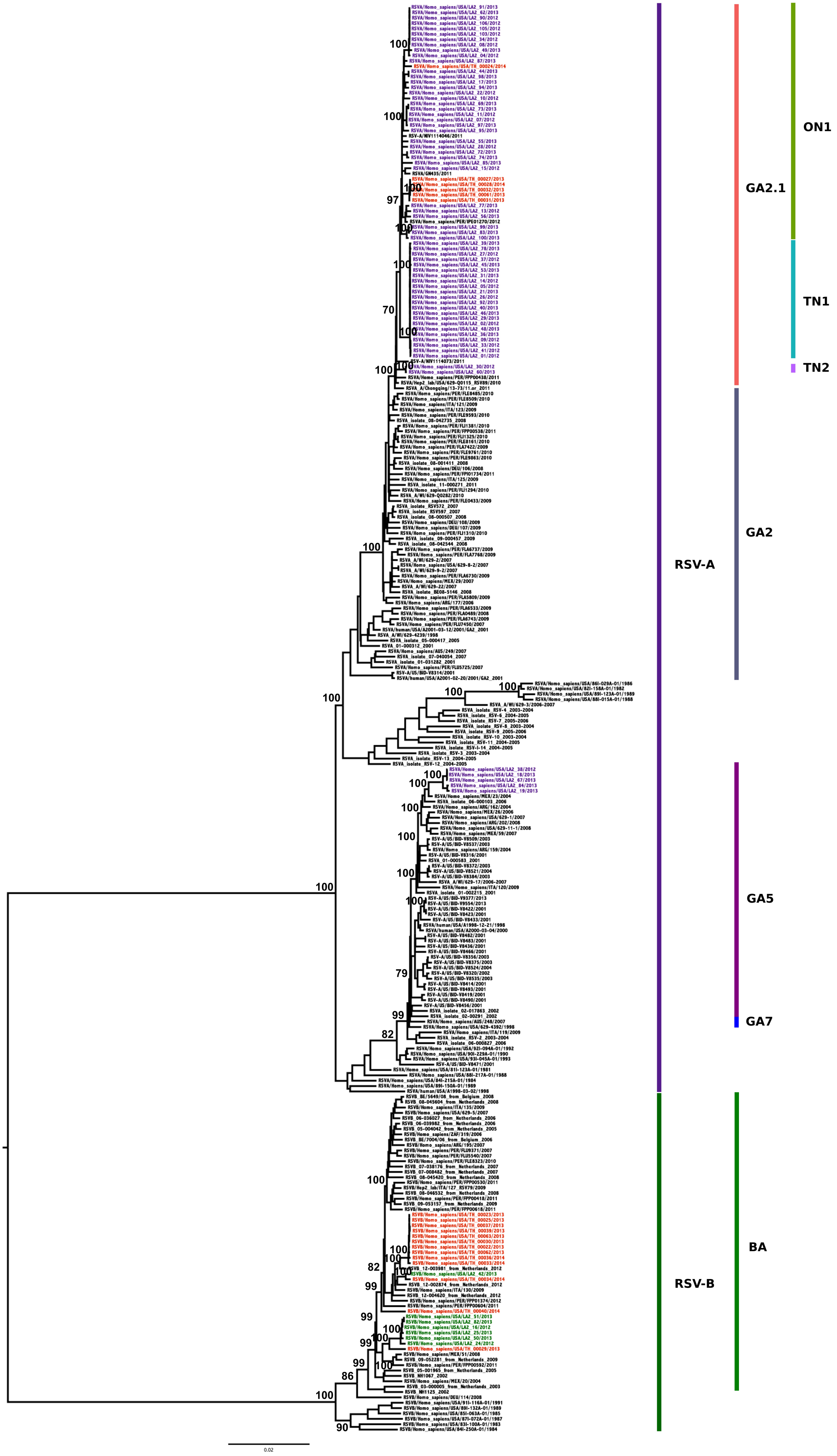

ON1
TN1
TN2
GA2.1
GA2
GA5
GA7
BA
RSV-A
RSV-B
0.02
